# Quantification of Heterogeneity as a Biomarker in Tumor Imaging: A Systematic Review

**DOI:** 10.1371/journal.pone.0110300

**Published:** 2014-10-20

**Authors:** Lejla Alic, Wiro J. Niessen, Jifke F. Veenland

**Affiliations:** 1 Biomedical Imaging Group Rotterdam, Department of Radiology and Medical Informatics, Erasmus Medical Center Rotterdam, Rotterdam, The Netherlands; 2 Department of Intelligent Imaging, Netherlands Organization for Applied Scientific Research (TNO), The Hague, The Netherlands; 3 Imaging Physics, Faculty of Applied Sciences, Delft University of Technology, Delft, The Netherlands; Yale University, United States of America

## Abstract

**Background:**

Many techniques are proposed for the quantification of tumor heterogeneity as an imaging biomarker for differentiation between tumor types, tumor grading, response monitoring and outcome prediction. However, in clinical practice these methods are barely used. This study evaluates the reported performance of the described methods and identifies barriers to their implementation in clinical practice.

**Methodology:**

The Ovid, Embase, and Cochrane Central databases were searched up to 20 September 2013. Heterogeneity analysis methods were classified into four categories, i.e., non-spatial methods (NSM), spatial grey level methods (SGLM), fractal analysis (FA) methods, and filters and transforms (F&T). The performance of the different methods was compared.

**Principal Findings:**

Of the 7351 potentially relevant publications, 209 were included. Of these studies, 58% reported the use of NSM, 49% SGLM, 10% FA, and 28% F&T. Differentiation between tumor types, tumor grading and/or outcome prediction was the goal in 87% of the studies. Overall, the reported area under the curve (AUC) ranged from 0.5 to 1 (median 0.87). No relation was found between the performance and the quantification methods used, or between the performance and the imaging modality. A negative correlation was found between the tumor-feature ratio and the AUC, which is presumably caused by overfitting in small datasets. Cross-validation was reported in 63% of the classification studies. Retrospective analyses were conducted in 57% of the studies without a clear description.

**Conclusions:**

In a research setting, heterogeneity quantification methods can differentiate between tumor types, grade tumors, and predict outcome and monitor treatment effects. To translate these methods to clinical practice, more prospective studies are required that use external datasets for validation: these datasets should be made available to the community to facilitate the development of new and improved methods.

## Introduction

Tumors are often inhomogeneous. Regional variations in cell death, metabolic activity, proliferation and vascular structure are observed. There is increasing evidence that solid tumors may consist of subpopulations of cells with different genotypes and phenotypes [1]. These distinct populations of cancer cells can interact in a competitive way [2] and may differ in sensitivity to treatments [3], [4]. This heterogeneity can be detected using diagnostic imaging techniques at a genetic, molecular or cellular level [4], [5], or at a cell population level. The advantage of diagnostic imaging techniques is their non-invasive nature and the fact that the whole tumor is taken into account, whereas cellular diagnostic techniques are invasive and limited to a discrete set of tumor samples. Various imaging techniques are available to visualize the heterogeneity in tissue characteristics, such as necrosis, metabolic activity, cell density and vascularity. Observed heterogeneity in an image is a reflection of the phenotypic variation of the tumor and is reported to be associated with underlying gene-expression patterns [6].

Image heterogeneity can be quantified using a variety of texture analysis methods. As such, image heterogeneity is potential biomarker for tumor characterization, for response prediction and monitoring. Parameters in hot spots, as quantified with dynamic contrast-enhanced magnetic resonance imaging (DCE-MRI), are more relevant for monitoring tumor response than parameters averaged over the whole tumor [7]–[9]. When a region of the tumor is not well vascularized or is hypoxic, chemotherapy and radiotherapy are more likely to fail. The existence of poorly vascularized or hypoxic areas within a tumor is an important component of tumor radiation resistance and correlates with treatment failure [10]. In radiotherapy, the heterogeneity can be used to guide treatment [11], [12]: an ongoing trial is currently escalating the dose to the part of the tumor with high standardized uptake values [13]. Also for computed tomography (CT), image heterogeneity has prognostic value [6].

Several methods are available to quantify tumor heterogeneity from imaging data. Many studies have used histogram-derived features such as percentile values, standard deviation (SD) and enhancing fraction. However, these features do not take into account the spatial distribution of the intensity values. In contrast, texture methods take spatial information into account by quantifying the spatial variations in the images. Ideally, these methods are independent of the absolute signal intensities in the image. They provide additional and independent information (such as the average signal intensity) compared to histogram-derived measures. These methods result in features which can be considered to be imaging biomarkers providing information on the underlying tumor heterogeneity. Some of these features are related to image properties that are visually perceived by the radiologist, whereas others are more abstract [14].

By means of a systematic review, the aim of this study is to investigate the performance of different heterogeneity imaging biomarkers extracted from diagnostic tumor images for differentiation between tumor types, tumor grading, outcome prediction and treatment monitoring.

The following research questions were formulated:

- Which analysis methods are used to quantify heterogeneity or texture in tumor imaging, with the aim to differentiate between tumor types, tumor grading, outcome prediction and treatment monitoring?
- What are the reported performances of the different analysis methods? Is there a relation between performance and analysis method?

What is the potential clinical impact of the methods? Can the performance results be generalized? Is the performance evaluated in addition to established imaging biomarkers?

## Methods

### Data Sources and Search method

This review was performed in accordance with the PRISMA (Preferred Reporting Items for Systematic Review and Meta-Analyses) guidelines [15], with details summarized in Checklist S1. In January 2013 the study protocol was registered with the International Prospective Register of Systematic Reviews (Identification number: CRD42013003634) [16]. A systematic search was conducted in the databases of Medline, Embase, and Cochrane Central. The search was performed with the aid of an experienced librarian on September 20^th^ 2013.

The following topics were used for the searches:

1. Neoplasms
2. Heterogeneity, texture
3. MRI, MRS, CT, PET, SPECT, ultrasonography
4. Differentiation between tumor types, tumor grading, classification, staging, treatment response, survival, and treatment outcome

Full details of the Embase search is included in Text S1. The results from all three searches were combined and verified to ensure exclusion of publications containing the same title, written by the same authors, and published in the same journal. The remaining publications were considered for study selection.

### Study Selection

Two authors (L.A. and J.F.V.) independently reviewed the titles and abstracts. The selected publications then underwent full-text screening. During the title and abstract review, any discrepancies about study inclusion were resolved by full-text screening. Any discrepancies during the following stages were resolved by discussion. The bibliographies of seminal review papers [17]–[19] were reviewed to identify additional relevant articles.

### Inclusion and exclusion criteria

We included only publications related to diagnostic imaging which reported quantification of tumor heterogeneity or tumor texture with the goal to differentiate between tumor types, tumor grading, outcome prediction and tumor response monitoring. No restrictions were made based on location, type, stage or grade of malignancy. Prior to review, a decision was made to exclude any study with too few participants, i.e., for patient studies (n<10) and for animal studies (n<5). Therefore, all case studies, and studies with no information on the number of subjects, were excluded. In addition, all the following types of studies were excluded:

- publications based on non-tumor images;
- publications not based on quantitative assessment of heterogeneity or texture in images
- publications without one of the following goals: differentiation between tumor types, tumor grading, or outcome prediction or treatment monitoring;
- publications not based on in vivo studies (histology, phantom, ex vivo, synthetic data);
- publications describing non-original research (editorial, letter to the editor, review, meta-analysis, opinion publications).

### Data extraction

A data extraction form was designed. All selected publications were independently reviewed and data extraction was cross-checked. Disagreements between the reviewers were resolved by consensus. The following data were extracted from the full papers: year of publication, human or animal study, type of study (retrospective or prospective), number of subjects, number of tumors, location of tumor, imaging modality, tracer/contrast agent, goal of heterogeneity/texture analysis, and type of heterogeneity/texture quantification method used. For studies reporting on the same analysis method based on the identical dataset, only the latest publication was included. For publications reporting classification experiments, the following data were extracted: number of candidate heterogeneity features, dimensionally reduction technique used, number of selected features used in the best classification experiment, the results of the best classification experiment, i.e., accuracy, sensitivity, specificity, area under the receiver operator curve (AUC), type of cross-validation used, and use of an external validation set. For publications using statistical hypothesis testing the following data were extracted: the number of candidate features, and the number of features that showed a significant difference between outcome categories (before and after Holm-Bonferroni correction) [20]. All publications were divided into two categories:

- Publications reporting cross-sectional measurements with the aim to differentiate between tumor types, tumor grading, and treatment outcome prediction.
- Publications reporting longitudinal measurements for tumor treatment monitoring.

### Data synthesis and analysis

The imaging modalities were summarized into four categories: i) magnetic resonance imaging (MRI), ii) computed tomography (CT), iii) positron emission tomography (PET), single photon emission computed tomography (SPECT), and iv) ultrasonography (US). No further subdivision was made regarding the type of imaging protocol or use of contrast agent.

Image analysis methods to estimate tumor heterogeneity were divided into four categories: non-spatial methods, local spatial distribution methods, fractal analysis, and a category consisting of filters and transforms.

#### Non-spatial methods (NSM)

These methods characterize tumor heterogeneity by non-spatial descriptors, such as descriptors of the gray-level frequency distributions: standard deviation, skewness, maximum, minimum, range, peak height, peak position, and percentile values.

#### Spatial gray-level methods (SGLM)

Methods included in the second category extract the local spatial image intensity distribution. This category includes grey-tone spatial-dependence matrix (GTSDM) [21], neighborhood gray-tone difference matrix (NGTDM) [22], run-length matrix (RLM), and Local Binary Pattern (LBP) [23]. The GTSDM, originally proposed by Haralick et al. [21], is often referred to as co-occurrence or the second-order histogram. When divided by the total number of neighboring pixels in the image, this matrix becomes the estimate of the joint probability of two pixels at a distance along a given direction having a particular gray value. The NGTDM, originally proposed by Amadasm and King [22], is based on spatial changes in gray values by inspecting the difference between gray levels of a specific pixel and the average gray level of their surrounding neighbors. The RLM, originally proposed by Galloway [24], is subsidiary to the observation that a coarse texture would have relatively longer gray level runs compared to a fine texture. This matrix provides information about runs of pixels with the same gray level values in a given direction. LBP, originally proposed by Ojala et al. [25] and later modified to a rotation and scale invariant approach [23], represents local texture. In its simplest form it labels the pixels of an image by thresholding the neighborhood of each pixel and considers the result as a binary number.

#### Fractal analysis (FA)

The third category consists of FA methods that overcome the scale problem by providing a statistical measure reflecting pattern changes as a function scale. The two basic parameters in FA are fractal dimension (FD) and lacunarity [26]. An often used method to estimate FD is box counting [26]. This procedure systematically overlays an image with a series of grids with increasing/decreasing size. For each step, this procedure captures the predefined relevant features [27]. Another frequently used technique in FA is the blanket method [26], which is often used in its extended form, as described by Peleg et al. [28]. This method estimates the surface area by measuring the volume between an upper and lower blanket.

#### Filters and Transforms (F&T)

The fourth category consists of a collection of image processing algorithms that extract texture features. Examples are methods that use techniques defined in the spatial domain such as filters (Gabor filters or Law’s filters) or transformations to other domains (Fourier transform, Wavelet transform, S-transform, discrete cosine transform). Since the various methods have only been used in a limited number of publications included in the present review, these methods were grouped together.

#### Publications reporting classification experiments

Publications were considered classification studies if they reported a classification result such as accuracy, sensitivity, specificity or AUC values. Only publications in which the results of the classification experiments were solely based on texture parameters were further analyzed. These studies often utilize a high number of candidate features to describe a tumor. When the number of extracted features is too large to perform a statistically meaningful classification [29], the extracted features can be redundant in the information they retain. Because an increase of dimensionality in the feature space results in an increase of its volume, the feature space is sparsely filled. The use of an extensive number of features for classification purposes can result in over-fitting, which reduces the possibility of generalization; this paradox is generally referred to as the ‘curse of dimensionality’ [30].

To keep the system manageable, dimensionality reduction techniques were commonly applied to select a subset of features that were relevant for the classification problem. The ratio between the number of tumors classified and the dimensionality of the feature space (e.g., the number of selected features) should be chosen in a meaningful way. In pattern recognition applications, the rule of thumb is to use 5–10 datasets per feature per category [31]. Therefore, we evaluated the number of candidate features, the number of selected features, and the ratio between the number of tumors included in the study and the number of selected classification features. A one-way ANOVA was used to test for differences in classification results between the modalities and analysis methods.

#### Publications reporting on significance testing

A commonly used approach to test the validity of the selected features is significance testing. For heterogeneity analysis, many publications compute a large number of features. As multiple comparisons generally require a stronger level of evidence to be considered significant, the Holm-Bonferroni correction [20] can be applied. This correction allows for the significance levels for single and multiple comparisons to be directly comparable. In these publications, we evaluated whether a Holm-Bonferroni correction was applied and, if this was not the case, computed the number of significant features after correction using the available data. A one-way ANOVA was used to test for differences in the number of significant features, before and after Holm-Bonferroni correction, between the modalities and the analysis methods used.

## Results

**Figure 1:**
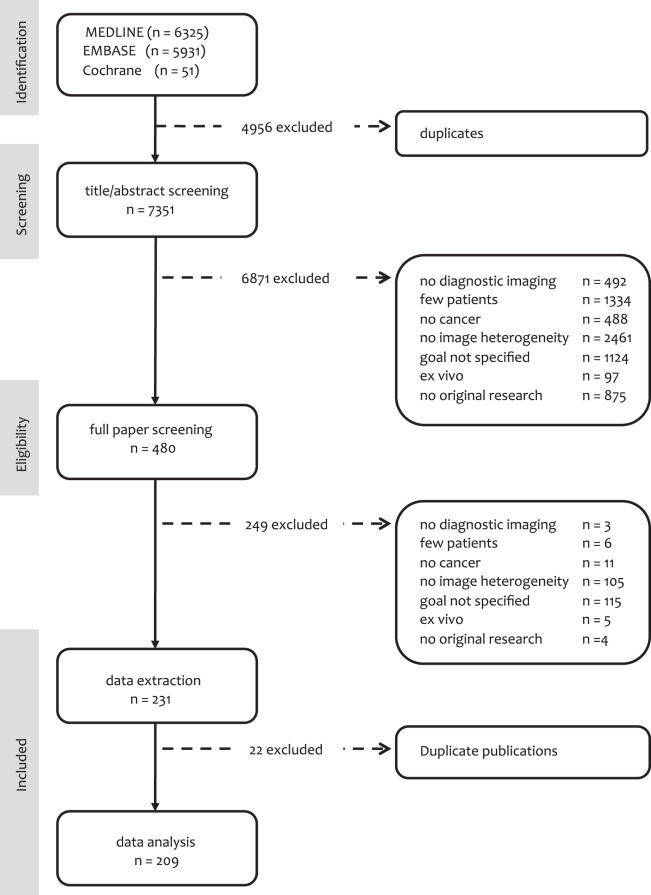
Results of the literature search. PRISMA flow diagram for study collection [15], showing the number of studies identified, screened, eligible, and included in the systematic review. This study is registered with the PROSPERO registry for systematic reviews (Identification number: CRD42013003634) [16].

Figure 1 presents details on the literature search. In summary, of the 7351 potentially relevant articles, 480 (6.5%) were considered for inclusion after abstract review. After these latter papers had undergone full-text screening, an additional 249 publications were excluded. The remaining 231 original publications entered the data extraction phase. In this phase an additional 22 papers [32]–[53] were excluded as they reported results of a similar analysis method on the same dataset as that used in another paper; for these publications, the most recent one was included in the analysis. Finally, data from 209 studies [7], [14], [54]–[228] were extracted for further analysis.

### General characteristics

Table 1 presents the characteristics of the included publications (after removing duplicate publications). A publication may include more than one imaging modality, analysis method, or goal. Two studies (1%) reported on two imaging modalities, and 66 studies (32%) reported on two or more analysis methods.

**Table 1:** Characteristics of the included publications (n = 209).

Characteristic		n	%
Imaging method	MRI	75	36%
	CT	40	19%
	PET	14	7%
	US	81	39%
Analysis method	NSM	121	58%
	SGLM	103	49%
	FA	21	10%
	F&T	58	28%
Study goal	Diagnosis/grading/outcome pred.	182	87%
	Response monitoring	27	13%
Study type	Retrospective	118	56%
	Retrospective (with inclusion criteria)	63	30%
	Prospective	28	13%
Type of subjects	Human	197	94%
	Animal	12	6%
Type of experiment	Classification	139	67%
	Significance testing	64	30%
	Neither	6	3%

Imaging modalities: magnetic resonance imaging (MRI), computed tomography (CT), positron emission tomography (PET), ultrasonography (US).

Analysis methods: non-spatial methods (NSM), spatial grey level methods (SGLM), fractal analysis (FA) methods, and filters and transforms (F&T).

**Figure 2:**
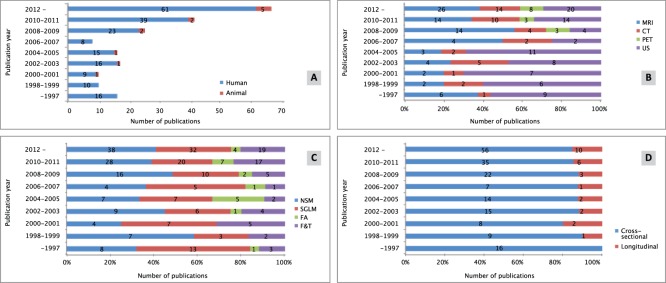
Number of publications reporting on tumor heterogeneity analysis for all publications bi-annually. Total number of publications (A), publications per imaging modality (B), publications per analysis method (C), and publications per goal (D).

Since 2008, the number of imaging studies quantifying tumor heterogeneity has been steadily increasing, i.e. from 8 papers in 2006–2007 to 66 publications in 2012–2013 (figure 2-A). Prior to 2006, heterogeneity was mainly studied based on US data (Figure 2-B). Since 2007, most studies quantifying tumor heterogeneity are based on MRI. Generally, the non-spatial method (NSM) and the spatial gray-level method (SGLM) are the most frequently used to analyze tumor heterogeneity (Figure 2-C). Although the number of publications using these methods has increased since 2007, their contribution to heterogeneity literature is relatively stable. The number of studies reporting tumor response monitoring has varied over the years, ranging from 0–20% (Figure 2-D).

**Figure 3:**
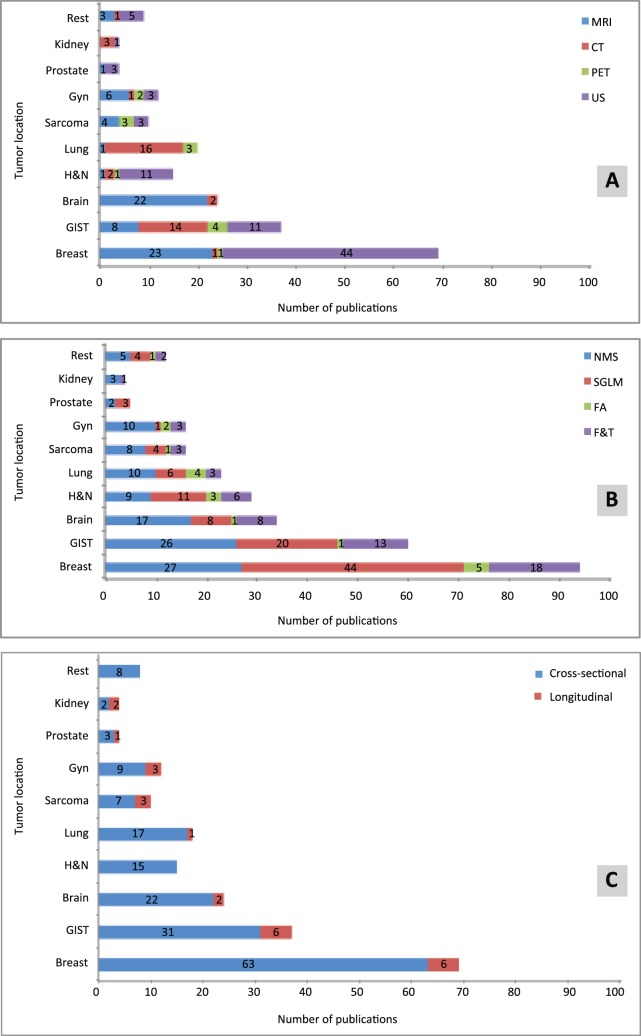
Publications reporting on quantification of tumor heterogeneity in cancer sites summarized for imaging modality (A), analysis method (B), and study aim (C). Publications can report on more than one analysis method. The acronyms used: Gyn – gynecological, H&N - head and neck, GIST – gastrointestinal.

Breast tumors were studied in 33% (n = 69) of the publications. Figure 3 shows the distribution of studies per tumor location. Figure 3-A shows the use of imaging modalities for quantification of tumor heterogeneity per primary tumor location. MRI is used primarily for brain and breast tumors, CT for lung and gastrointestinal tumors, PET for gastrointestinal, lung tumors and sarcoma, and US for breast tumors. Heterogeneity analysis of brain tumors was performed almost exclusively with MRI, while for breast tumors both MRI and US were used.

Figure 3-B presents the analysis methods used per primary tumor location. For almost all locations, all methods were used. For prostate, breast, and head and neck analysis, the SGLM was the most frequently used. For all other locations, the NSM was the favored modality. Heterogeneity analyses for longitudinal studies were mainly performed for gastrointestinal and breast tumors (Figure 3-C).

Figure S1 summarizes the publications included in the present review (n = 209) in a matrix form. The publications are divided into different imaging modalities and analysis methods, and are available for download for each cell separately. Each cell in the matrix links to the supplementary EndNote file containing the records for these publications.

**Figure 4:**
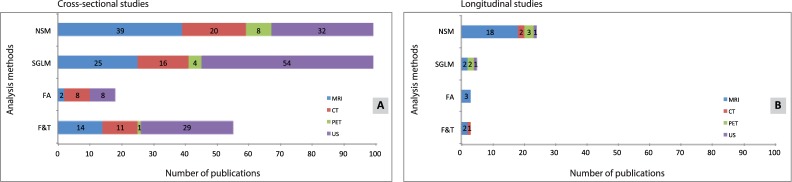
All included publications reporting cross-sectional (A) and longitudinal (B) studies. Several publications report more than one analysis method.

Figure 4-A shows the relation between imaging modality and analysis methods for cross-sectional studies. In general, 74% of these studies used either MRI or US. The SGLM (37%) and NSM (36%) are most frequently used to grade and diagnose tumors. Figure 4-B shows the relation between imaging modality and analysis method for the longitudinal studies (n = 27). MRI was used in 70% of these studies and PET in 11%. In 7% of the studies, US-based heterogeneity quantification was used for tumor response monitoring. NSM is the most frequently used (69%) analysis method in longitudinal studies.

**Figure 5:**
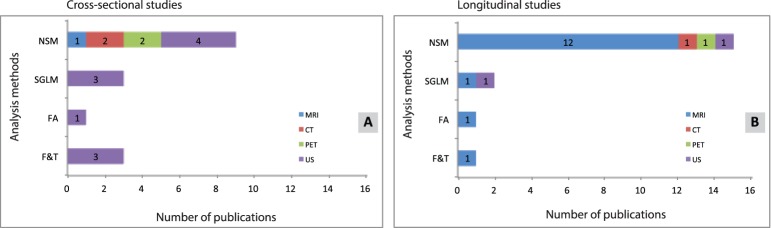
Publications reporting a prospective study design cross-sectional (At) and longitudinal (B) studies. Several publications report more than one analysis method.

A relatively small number of all studies (13%) utilized a prospective study design. Figure 5-A shows the relation between imaging modality and analysis method used for cross-sectional studies (n = 12). US is the most frequently used modality, whereas NSM is the most frequently used analysis method. Figure 5-B shows the relation between imaging modality and analysis method for publications reporting longitudinal studies (n = 16). Again, most data were analyzed with NSM. In contrast to MRI, CT, US and PET are rarely used for heterogeneity quantification in prospective longitudinal studies.

### Publications reporting classification experiments

Of all included studies, 67% (n = 139) reported classification experiments and 30% reported significance testing. The remaining 3% either did not report quantitative results or the experiments were not completely described. Also, 23 studies only reported results of classification experiments where the texture features were combined with non-texture features. For these latter publications, it was not possible to extract the performance of the texture features separately and, therefore, these results were excluded from further analysis. Additionally, 10 studies were excluded because the number of generated or selected features was lacking. Of the papers reporting classification experiments (n = 106), 45% used US, 37% used MRI, 13% used CT, and 5% used PET. In 42% of the classification papers, features originating from different texture analysis methods were combined. Some studies reporting classification experiments (n = 39) performed no feature reduction, and the median number of candidate features used in these studies (6) was significantly lower than that of candidate features in the studies using feature reduction techniques (38). The remaining 67 studies reporting classification experiments used one of the methods commonly applied in statistics, pattern recognition, or machine learning. These methods were summarized into three categories: filters, wrappers and embedded methods [229].

**Figure 6:**
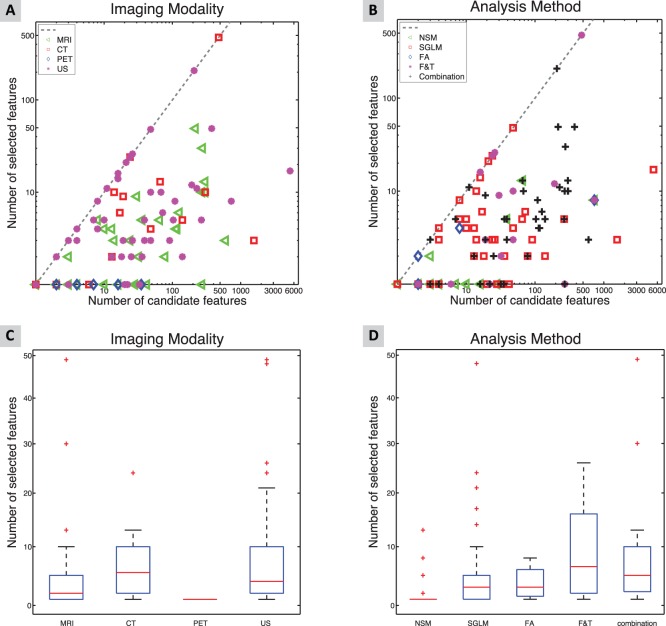
Number of features used in classification experiments for different imaging modalities (A) and for different analysis methods (B). Boxplot representing distribution in number selected features for imaging modality (C) and for analysis methods (D). To enhance visibility, we excluded for both boxplots two studies with large numbers of selected features.

Figure 6 shows the relation between the number of candidate features and the number of selected features used in classification experiments for different imaging modalities (Figure 6-A) and different analysis methods (Figure 6-B). For the papers presented on the dotted line, no feature selection was performed. The number of candidate features ranged from 1–5280 (median 22) while the number of selected features ranged from 1–476 (median 3). The distribution of the numbers of selected features can be assessed as boxplots for imaging modality (Figure 6-C) and for analysis methods (Figure 6-D).

About 63% of the publications describing a classification experiment, reported cross- validation or training test sets as a technique to limit the effect of over-fitting on the available data. Figure 6-B shows that the combination of features from different methods generally leads to a higher number of candidate features. In general, in publications reporting the use of more than one analysis method more extensive feature reduction is applied compared to publications reporting on the use of the separate analysis methods.

**Figure 7:**
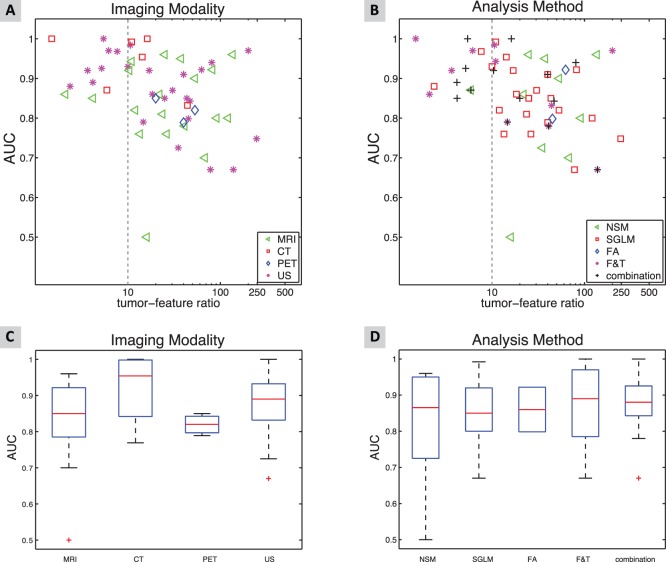
The AUC for different imaging modalities (A) and for different analysis methods (B) as a function of tumor-feature ratio in the classification experiments. The scatter plot shows each imaging modality and analysis method separately. Dotted line represents the ratio of 10 tumors per selected feature. Boxplot representing distribution in AUC for imaging modality (C) and for analysis methods (D).

In the classification experiments, one or more of the following performance measures were reported: sensitivity, specificity, accuracy, or AUC. Figure 7-A shows the AUC per imaging modality and Figure 7-B the AUC per analysis method. The differences in performance (as measured by AUC) are shown in Figure 7-C per imaging modality and in Figure 7-D per analysis method.

The supplementary material provides the figures for accuracy (Figure S2), sensitivity (Figure S3) and specificity (Figure S4) per imaging modality and per analysis method. In these figures, the reported performance is depicted as a function of the tumor-feature ratio (ratio between the number of tumors included and the number of selected features). In general, the tumor-feature ratio ranged from 0.46–502 (median 20) with (on average) 29% of the publications showing a tumor-feature ratio ≤10.

With respect to the analysis method, publications using the F&T, or a combination of methods, had the highest risk of a tumor-feature ratio ≤10, i.e. 53% and 42%, respectively. With regard to imaging modality, CT publications had the highest percentage (43%) with a tumor-feature ratio <10.

Using a one-way ANOVA, no significant differences were found in the performance measures between the modalities or between the analysis methods used. However, there was a negative correlation between the logarithm of the number of tumors per selected feature and the AUC (r = −0.32, p<0.05) and the specificity (r = −0.48, p<0.05).

### Publications using statistical hypothesis testing

**Figure 8:**
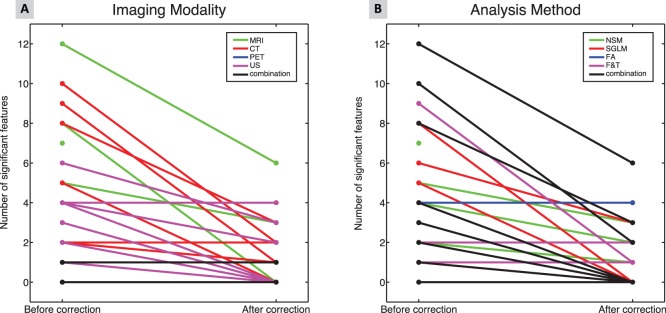
Number of significant features before and after Holm-Bonferroni correction in publications reporting on significance testing for all image modalities (A) and all analysis methods (B).

Of all included studies, 30% (n = 64) reported statistical hypothesis testing with the number of features ranging from 1–320 (median 4). Of these studies, 39% were based on MRI, 26%, on CT, 14% on PET, and 21% on US. Similarly, in 61% of the cases, data were analyzed using NSM, 12% using SGLM, 3% using FA, 6% using F&T, and 18% using a combination of these methods. The number of significant features, as reported by the authors, ranged from 0–76 (median 1). Since multiple comparisons generally require a stronger level of evidence to be considered significant, the Holm-Bonferroni correction [20] was applied by the original research authors, or by the authors of this review paper. This correction allows direct comparison to be made of the significance levels of single and multiple comparisons. For eight papers the correction could not be performed due to missing information. After the Holm-Bonferroni correction, the number of significant features ranged from 0–6 (median 1). Figure 8 shows the number of significant features before and after the Holm-Bonferroni correction per imaging modality (A) and per analysis method (B). In 45% of the papers the number of significant features decreased after correction. Using a one-way ANOVA, no significant differences were found in the number of significant features between the modalities. With respect to the analysis method used, a one-way ANOVA established a significant difference in the number of significant features (p<0.018). Publications using SGLM reported more significant features. However, after the Holm-Bonferroni correction, the numbers of significant features were similar between all analysis methods used.

## Discussion

This systematic review investigated the use and performance of heterogeneity or texture quantification methods in radiological images for differentiation between tumor types, tumor grading, outcome prediction and treatment response monitoring. After a systematic literature search yielding 7351, 209 unique studies reported on heterogeneity as an imaging biomarker in tumor imaging. Since 2008, an increasing number of publications have reported on quantification of tumor heterogeneity. Since the present review is based on the existing literature, it reflects the modalities, heterogeneity analysis methods, and location of tumors that were investigated by the authors of the included studies. Because almost all of the included publications presented positive results, it should be noted that this literature probably contains an over presentation of modalities, heterogeneity analysis methods and tumor locations for which heterogeneity analysis seems to work.

Until 2006 most heterogeneity papers were based on US, whereas after 2007 there was an increase in the number of studies using MRI. During the present study period, NSM and SGLM were the most frequently used methods. Most of the papers focus on heterogeneity quantification to differentiate between tumor types, tumor grading or outcome prediction; however, the number of papers with the goal of response monitoring has recently increased. In tumor heterogeneity quantification, US is the most frequently used imaging modality for differentiation between tumor types, tumor grading and outcome prediction, and MRI is the most frequently used modality for treatment response monitoring. For monitoring of treatment response, NSM is the most frequently used method. To differentiate between tumor types and tumor grading, all methods are evenly distributed over all the modalities.

The performance of the heterogeneity features was mostly (67%) evaluated by classification experiments reporting performance measures such as accuracy, sensitivity, specificity and AUC. Papers reporting only on the results of the combination of texture features with other features were excluded from the analysis. Some authors selectively report on sensitivity without mentioning the specificity. The AUC is the preferred measure to report performance as it is more comprehensive compared to a measure based on a single threshold, such as accuracy. Only one paper reported an AUC of 0.5, all other papers reported higher values. This is most likely caused by publication bias: only the positive performance of heterogeneity features tend to reach the journals. Only 63% of the publications reporting classification results described the use of the cross-validation technique to limit the effect of over-fitting on the available data. We found no relation between the performance measures and the modality, or with the analysis method used. However, a negative correlation was found between the tumor-feature ratio and the AUC. When more tumors were available per selected feature, the AUC was lower. This correlation may be the result of overfitting of the data when fewer tumors per feature are available.

Publications using statistical hypothesis testing often did not perform a correction of the significance levels for multiple comparisons. For eight papers, due to missing information, a retrospective Holm-Bonferroni correction could not be performed by the authors. For 45% of the papers, the number of significant features decreased after the Holm-Bonferroni correction. We found no relation between the number of significant features after the Holm-Bonferroni correction and the modality or the analysis method used.

The number of prospective studies is small, i.e. only 13% of all studies. These latter studies are mainly based on MRI and report NSM features. Although the use of retrospectively collected data is necessary to develop, test and evaluate heterogeneity as a biomarker for differentiation between tumor types, tumor grading, outcome prediction and treatment response monitoring, the real test is to evaluate the performance of the developed features in a prospective study design. When using a retrospective study design, the criteria for the inclusion of cases are often not (or not clearly) described, so that the performance of the heterogeneity feature can be overestimated. Using a prospective study design, with clear inclusion criteria, the actual performance of heterogeneity features can be more reliably assessed.

Moreover, in most included studies, performance of the heterogeneity feature is evaluated without taking into account currently accepted clinical features, such as mean signal intensity, tumor size, tumor grade, or border regularity of a tumor. Some studies report only the combined classification performance of heterogeneity and clinical features. A large number of publications even use the mean signal intensity as a feature to estimate tumor heterogeneity, even though this is clearly not a heterogeneity measure (i.e., mean signal intensity does not measure intra-tumor heterogeneity). Based on these types of studies, it is not possible to evaluate the added value of heterogeneity to currently accepted clinical features. Whereas researchers are interested in the performance of the feature itself, clinicians are interested in the additional value of the feature compared with the currently available clinical biomarkers. Since the quantification of heterogeneity is usually more complex and computationally more costly than computing the mean intensity, the benefit of the added effort to characterize heterogeneity needs sufficient motivation. To enable the translation of imaging biomarkers from the research stage to clinical practice, future research should focus on studies investigating the additional value of the proposed heterogeneity biomarker compared with the established clinical markers.

In this systematic review, comparison between the performance of different methods for a certain classification task was not possible due to the large variety in the datasets used and the classification tasks posed. The search for new and optimal (combinations of) heterogeneity features would benefit from developing reliable datasets (for different classification problems) that are available to the scientific community. Large well-defined datasets are a prerequisite for objective comparison of methods.

Future studies should have a design that takes the requirements from pattern recognition into account, i.e. a balanced number of subjects and features, cross-validation, independent test datasets, and a prospective study design. Satisfying these requirements will allow more reliable evaluation of the value of heterogeneity features.

## Supporting Information

Figure S1 Numbers of publications for a specific imaging modality and analysis method. The supplementary EndNote files corresponding to the records for these publications (for each cell in the matrix separately) are publically available. To download separate files just click on a cell of interest in the figure.
(PDF)

Figure S2 The accuracy for different imaging modalities (A) and for different analysis methods (B) as a function of tumor-feature ratio in the classification experiments. The scatter plot shows each imaging modality and analysis method separately. Dotted line represents the ratio of 10 tumors per selected feature. Boxplot representing distribution in AUC for imaging modality (C) and for analysis methods (D).
(EPS)

Figure S3 The sensitivity for different imaging modalities (A) and for different analysis methods (B) as a function of tumor-feature ratio in the classification experiments. The scatter plot shows each imaging modality and analysis method separately. Dotted line represents the ratio of 10 tumors per selected feature. Boxplot representing distribution in AUC for imaging modality (C) and for analysis methods (D).
(EPS)

Figure S4 The specificity for different imaging modalities (A) and for different analysis methods (B) as a function of tumor-feature ratio in the classification experiments. The scatter plot shows each imaging modality and analysis method separately. Dotted line represents the ratio of 10 tumors per selected feature. Boxplot representing distribution in AUC for imaging modality (C) and for analysis methods (D).
(EPS)

Text S1 Comprehensive EMBASE search strategy used in the systematic review.
(PDF)

Checklist S1 PRISMA checklist for the systematic review: Quantification of heterogeneity as a biomarker in tumor imaging.
(PDF)
