# Supplementary figures and images for: Quantification of Heterogeneity as a Biomarker in Tumor Imaging: A Systematic Review
Source: PLoS One. 2014 Oct 20;9(10):e110300. doi: 10.1371/journal.pone.0110300 (PMC4203782; doi:10.1371/journal.pone.0110300)

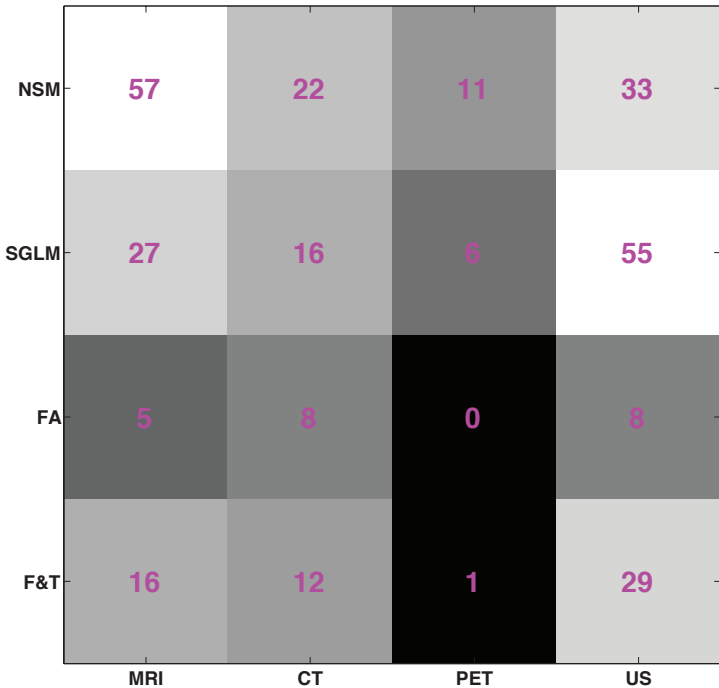

Supplement: Figure S1 — Numbers of publications for a specific imaging modality and analysis method. The supplementary EndNote files corresponding to the records for these publications (for each cell in the matrix separately) are publically available. To download separate files just click on a cell of interest in the figure. (PDF) [file pone.0110300.s001.pdf]
