# Supplementary material for: Quantification of Heterogeneity as a Biomarker in Tumor Imaging: A Systematic Review
Source: PLoS One. 2014 Oct 20;9(10):e110300. doi: 10.1371/journal.pone.0110300 (PMC4203782; doi:10.1371/journal.pone.0110300)
Supplement: Text S1 — Comprehensive EMBASE search strategy used in the systematic review. (PDF) [file pone.0110300.s005.pdf]

Lejla Alic

Quantification of heterogeneity as a biomarker in tumour imaging: a systematic review

EMBASE search

('neoplasm'/exp OR (neoplasm* OR cancer* OR tumour* OR tumor* or malign* or carcinoma*):ab,ti) AND (textur* OR heterogen* OR homogen* OR inhomogen*):ab,ti AND (analy* OR method* OR feature* OR characteri* OR quantif* OR quantitat* OR signature* OR marker* OR parameter*):ab,ti AND ('nuclear magnetic resonance imaging'/exp OR ‘computer assisted tomography’/exp OR 'emission tomography'/exp OR echography/exp OR (mri OR 'magnetic resonance' OR 'mr imaging' OR ct OR tomograph* OR pet OR spect OR ultraso* OR echogra* OR sonogra* OR ‘single photon’):ab,ti) AND ('treatment response'/de OR survival/exp OR 'treatment outcome'/exp OR staging/de OR 'tumor classification'/exp OR 'differential diagnosis'/de OR 'tissue differentiation'/de OR 'tissue characterization'/de OR (response* OR surviv* OR monitor* OR outcome* OR classif* OR staging* OR differentia* OR distinguish* OR grad* OR characteri* or discrimin*):ab,ti)
